# Global mental health: perspectives from Ethiopia

**DOI:** 10.3402/gha.v7.25447

**Published:** 2014-09-30

**Authors:** Abebaw Fekadu, Graham Thornicroft

**Affiliations:** 1 Department of Psychiatry, School of Medicine, College of Health Sciences, Addis Ababa University, Addis Ababa, Ethiopia; 2 Department of Psychological Medicine, Institute of Psychiatry, Centre for Affective Disorders, King's College London, London, UK; 3 Health Services and Population Research Department, Institute of Psychiatry, King's College London, London, UK

**Keywords:** global mental health, health service, treatment gap, Ethiopia

## Abstract

**Background:**

Global mental health (GMH) advocates for access to and the equitable provision of mental health care. Although the treatment gap is a useful construct to measure access and equitability of care, it fails to communicate the real-life consequences of the treatment gap and the urgent need to address care disparities.

**Objective:**

The aim of this article is to present a perspective on the practical application of the principles of GMH to understand the real-life impact of the treatment gap and the approaches taken to improve treatment coverage in Ethiopia.

**Design:**

A case study method is used.

**Results:**

Multiple international collaborations undertaken in Ethiopia and facilitated by GMH to improve care, capacity, and the evidence base for increased treatment coverage are described briefly. A series of steps taken at the local and national levels to address the treatment gap are highlighted. The stories of two patients are also presented to illustrate the real-life consequences of the treatment gap and the potential transformational impact of addressing the treatment gap on patients, families, and communities.

**Conclusions:**

GMH has a key role to play in addressing the treatment gap, which improves the life of people with mental disorders, their families, and their communities. However, national-level policy support and coordination are essential for any realistic improvement in treatment coverage. The reflections offered through the case examples may have utility in similar low-income settings.

Global mental health (GMH) may be defined narrowly as a subset of global health (1) that advocates for an interdisciplinary and global effort to fully provide treatment for people with mental disorders and to decrease disparities in care. GMH provides three clear insights that have transformed the debate about mental disorders in low- and middle-income countries (LMICs). First, GMH has assembled a consistent body of evidence associating mental disorders with substantial societal burden (2). Even in settings where the burden from infectious and nutritional conditions remains overwhelming, at least 9% of the overall burden of disease is attributable to mental disorders (3). Second, GMH has documented that effective interventions exist for the large majority of mental disorders (4). Third, the proportion of people with mental illness who receive effective, evidence-based care is very low; thus, the treatment gap remains unacceptably high (5, 6). Globally, about 50% of people with psychosis may not receive good-quality care (7), but in countries like Ethiopia this proportion may well be over 90% (8, 9).

The implication of this huge treatment gap in LMICs is sobering. Very many people with severe mental illnesses, such as psychosis, are kept in chains and hidden away for years. These patients are often the victims of stigma and discrimination, invariably unproductive, and often physically and mentally abused (10, 11). Such neglect appears to be greater in rural settings (12); stigma also often adversely affects family members (13).

Applied practically in a LMIC setting like Ethiopia, GMH should provide data to demonstrate the impact of the treatment gap and a contextualised framework or approach to help reduce it. The aim of this article is therefore to illustrate the real-life consequences of the mental health treatment gap in LMICs with a case example from a rural area in Ethiopia, and then to outline positive responses and recent developments at the local (district) and national levels to address the treatment gap – lessons that may have resonance in other low-income settings worldwide. We chose Ethiopia as a case example primarily because we have more intimate knowledge of Ethiopia than other LMICs. But Ethiopia may also typify LMICs in terms of its developing economic status, restructuring, population growth and structure, urbanisation, and health needs.

## Methods

We employed the case study method, which makes use of both quantitative and qualitative approaches. The method is considered ideal when multiple interventions are involved which cannot be readily aggregated and when outcomes are long term and likely to be overdetermined (14). The case study method allows researchers to be guided by what is observed in the field; it also provides new facts that can inform assumptions, hypotheses, and further refinement of procedures (15). Within this framework, we use two stories of patients to demonstrate the impact of the treatment gap and the transformative impact of simple treatments. We describe the case of the Butajira project, a research study that also provided care to patients. Additionally, we put together what we have observed in Ethiopia as witnesses and participants of the effort to improve treatment coverage and research in the country.

## Results

### The personal consequences of the mental health treatment gap

The following example illustrates the impact that lack of accessible care had for one woman and her family in Ethiopia.

**Fig. 1:**
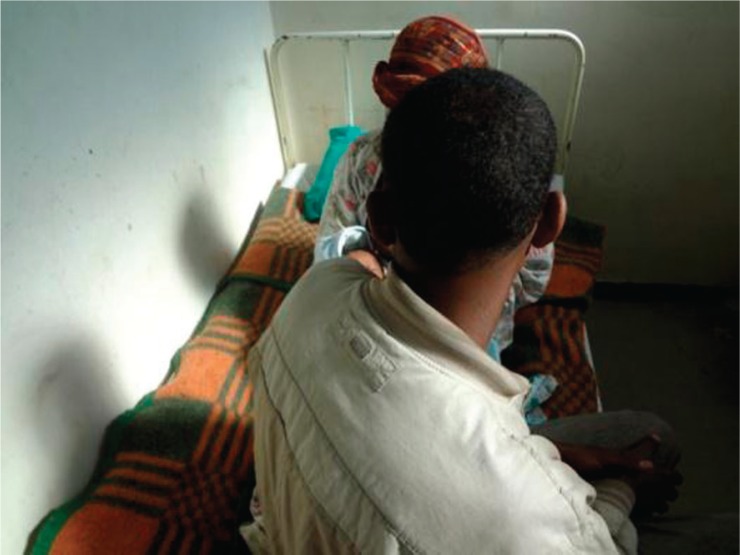
A picture of the couple with their 3-week-old baby boy in a psychiatric hospital bed in Addis Ababa. Photo by Abebaw Fekadu.

*Case Example 1:* The couple shown in Fig. 1 are farmers from a small rural village in southern Ethiopia. When Mrs. A. found out that she was pregnant after 6 years of marriage, the couple and their extended family were overjoyed. But their joy was short-lived. Mrs. A. developed a severe psychotic illness at 5 months of pregnancy and had to be ‘chained’ and taken to local traditional healers. Her symptoms escalated quickly, and there was serious concern about her safety and the safety of the unborn baby. To get treatment, she had to take a 3-day journey (with her husband and brother) to Addis Ababa, where the only specialised mental hospital is found. At the end of a complicated journey and several days of waiting for a bed, Mrs. A was admitted to the general ward of the hospital. Her husband had to remain in Addis Ababa while she was in the hospital, forgoing his farming at the risk of not having any harvest for the following year. Fortunately, despite additional traumatic experiences while in the general ward, her condition improved and she gave birth to a healthy baby boy. She was completely well and was discharged after 4 months of inpatient care. The next major hurdle she faced was the same long, expensive journey back to Addis Ababa to continue her treatment and clinical follow-up, as there is no local service that could provide the care she needs. Unfortunately, she has not returned since her discharge around one year ago and the clinical team does not know what might have happened to her.

This is a familiar story. In Ethiopia, as in many low-income countries, only a very small percentage of patients, in the order of 5 to 10%, who require care that may be life-saving actually receive it (8, 16). This is the case not only for people with psychotic disorders but also for those with epilepsy. Both these conditions can be eminently treated at low cost (17, 18), but the rates of treatment coverage and retention in care for both conditions are stubbornly low (9).

### Improving local (district-level) treatment coverage

**Fig. 2:**
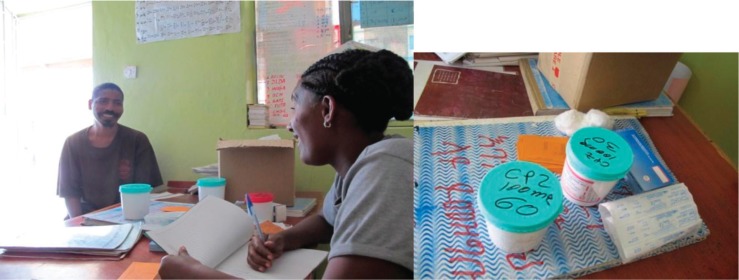
Psychiatric nurse at an outreach clinic in Butajira (showing the necessary equipment of case register, chlorpromazine, and fluphenazine depot). Photos by Abebaw Fekadu.

Coverage can be simply defined as the proportion of people with a given condition who receive appropriate treatment. The following example, the Butajira project (19), demonstrates that it is possible to implement a district mental health plan that substantially increases treatment coverage. The Butajira project was initiated 15 years ago, comprising both new district clinic services for people with mental illness and a programme of epidemiologic and implementation research. Although the primary reason for poor coverage is lack of accessible care, low demand and poor quality of care are also important barriers. The Butajira project implemented a ‘stepped-care’ model of service to address these barriers (Panel 1). Serving a total population of over two million, both an outpatient (ambulatory) mental health clinic and the only outreach programme in the whole country (allowing home visits) were provided to ensure accessible care. The project mobilised the community to generate demand and inclusiveness, and it ensured quality care by providing periodic supportive supervision and second-opinion clinics. Those who required further specialist care (mainly admissions) were supported to access a specialist service in Addis Ababa, although only four patients (of 919 adults in the cohort) required such specialist support. The mental illness research cohort for the Butajira study was identified after screening nearly 70,000 adults aged 15–49 years for severe mental illness, making this the largest such community-based study in Africa. At present, the clinic provides care for more than 7000 patients per annum, predominantly residing in the immediately neighbouring districts. The equipment needed for the outreach clinics is remarkably simple, as shown in Fig. 2. To illustrate further the impact of improved access through the Butajira project, a second case example is provided.

**Panel 1**

- The mental health treatment gap has a far-reaching impact on people and society that is not immediately apparent in discussions about global mental health or the treatment gap.
- Given the complexity of addressing the treatment gap, evidence applicable to the specific context should be sought, as exemplified by the experience in Ethiopia.
- Integrated services on their own are not adequate. The stepped-care model, which engages the community, integrates mental health care into primary care, and provision of specialist support is important.
  - Community engagement improves demand, supports community integration, and has the potential to improve quality of care.
  - Integrated care has the potential to improve accessibility of care as long as supervisory and quality control mechanisms are in place.
  - Mental health services should provide support to integrated care and provide services linked with the integrated care.
  - Services with specialist capacity to address complex needs should not be neglected.
- Governments and health ministries should provide leadership in meeting the treatment gap needs as exemplified in Ethiopia.

**Fig. 3:**
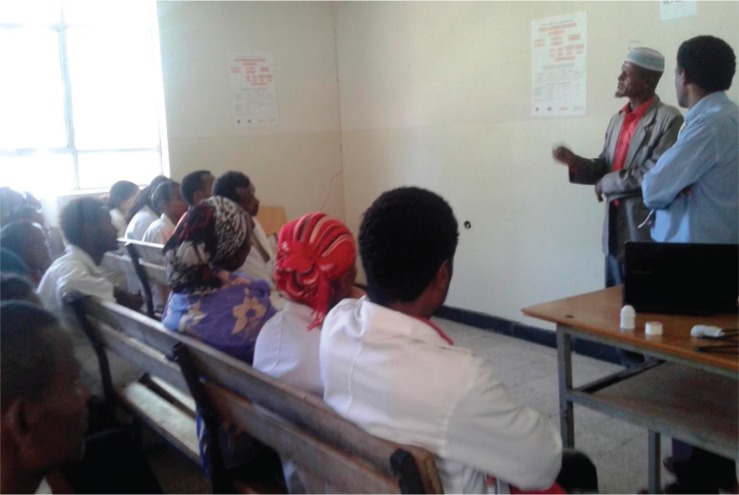
Mr B explaining about his illness and recovery to primary care staff during a training programme to initiate integrated care at the facility. Photo by Sr. Medhin Selamu.

*Case Example 2:* Mr B is currently the chair of an Idir – a traditional self-help community organisation for supporting members during times of difficulty, such as a death in the family – representing about 160 households. Four years ago, Mr B was a chair of a sub-district administration, where decisions of high impact relating to the residents are made. He is married with nine children, all in school or at work. He has a flourishing farmland, and owns the best house in the neighbourhood. But it was not always this way for Mr B. About 12 years earlier, he had developed a very serious psychotic illness. Because of his assaultive behaviour, he was chained for the better part of 4 years and deserted by his family and neighbours, with the exception of his wife, whom he now praises profusely. He was taken around to all known traditional healing sites in the area to no avail before he was found in chains by Butajira project field workers while the workers were making home visits. He was treated successfully by the project service and has since been well enough to lead not only his own family but also a sub-district of about 1,000 households. He was able to build his fortune and become a highly respected member of society. Mr B has been out of treatment for the past 2 years and attends mental health meetings and trainings to explain his recovery (Fig. 3).

Mr B is only one of a large number of individuals in the Butajira project for whom treatment by the psychiatric service of the project had made a fundamentally life-changing impact. From a situation in which he was chained, clearly violating the Convention on the Rights of Persons with Disabilities (20), he has regained his liberty, his family role, and his economic self-sufficiency. This is consonant with the overall results of the Butajira project, which found that treatment with psychotropic medication was associated with better outcomes and lower mortality rates for people with schizophrenia (21), bipolar disorder (22), and major depressive disorder (23). Indeed, these findings are consistent with the broader literature on the effectiveness of evidence-based treatments in LMICs (4). An evaluation of the project additionally found that the service was acceptable to local people and reduced family burden (24).

### National-level initiative to reduce the mental health gap

Until 1987, specialist psychiatric services in Ethiopia were centralised in the capital city of Addis Ababa and provided only at the university hospital and the specialised psychiatric hospital (Amanuel Psychiatric Hospital). A psychiatric nursing programme was then initiated with the support of the World Health Organization (WHO) (http://www.unspecial.org/UNS612/uns_612_T16.html). Trainee psychiatric nurses received 9 months of training that involved a 3-month taught course and 6 months of internship. For the internship, the trainees went back to their regions, established out-patient clinics, and were supervised by psychiatrists from Addis Ababa. The trained psychiatric nurses received periodic refresher courses, and the supervision was eventually phased out when it was believed that the psychiatric nurses could run the programme independently. The consequence was the expansion of specialised mental health services to 58 sites across the country, a major achievement for any service programme.

### The practical aspects of scaling up mental health care in Ethiopia

The improvement to mental health care in Ethiopia is a good example of gradually implementing changes that are designed to reduce the treatment gap by scaling up the provision of care in a sustainable way. The latest Health Sector Development Programme (HSDP-IV) (25) has set a target for integration of mental health into 50% of the healthcare settings by the end of the HSDP-IV in 2015. The recently launched National Mental Health Strategy (26) places ‘integrated care’ at its centre point, namely through training general primary care staff in the skills necessary to identify and treat people with mental illness. It specifically targets several priority disorders: psychosis, epilepsy, depression, and substance use disorders. The mental health strategy is considered to be a good template that could potentially be utilised by other countries in Africa (27). The strategy encourages the involvement of informal sectors to support mental health care in the community, specifically through the involvement of teachers, nongovernmental organisations (NGOs), and especially traditional and religious leaders. The strategy acknowledges that traditional healers and religious leaders account for a ‘significant extent of mental health care’ and so aims to provide them with ‘training’ to improve detection and referral. The informal-sector players are expected to work collaboratively with the formal sector. However, so far, there is limited communication between the formal and informal sectors, and it is unclear how collaboration could be initiated and made mutually reinforcing for the benefit of patients with mental illness. In this regard, an interesting experiment is being undertaken by the Department of Psychiatry at Addis Ababa University, in which the department runs a two weekly psychiatric clinic at a famous traditional healing site just outside of Addis Ababa. The service seems to have good acceptability, and there is a plan for evaluating the acceptability of the service and its impact.

Additionally, the financial management system has become more flexible so that the health care facilities and districts themselves have sufficient authority to make the necessary medical supplies and other resources available at the primary care centres.

The Mental Health Gap Action Programme (mhGAP) (9), a WHO-supported programme, has been successfully adapted to the situation in Ethiopia by the Ministry of Health. However, these policy initiatives are necessary but not sufficient. For such policy initiatives to take good effect and to be maintained in the long term, a series of health system strengthening measures are necessary. Several questions remain: for example, how does primary care integration work in practice? How can the health system support the necessary supervision, which is likely to be an important determinant of the success of an integrated programme? What should the roles of the community and the informal sector be? How could the health system support the well-being and morale of primary care staff in a feasible and scalable way? To address these issues, additional research on health services and health systems is being undertaken. For example, the Programme for Improving Mental Health Care (PRIME) (see http://www.prime.uct.ac.za), an international research and implementation programme funded by the UK Department for International Development, is now assessing the practical requirements of service integration and scale-up (28). The evidence base for redistributing clinical tasks from more senior staff, who are few on the ground, to those who are available in primary care settings is being investigated by the new Task Sharing for the Care of Severe Mental Disorders (TASCS) project, which is funded by the US National Institutes of Health. This study focusses on severe mental disorders (see http://www.affirm.uct.ac.za). Furthermore, the European Commission funded the Emerging mental health systems in low- and middle-income countries (EMERALD) project, putting into place a series of health system strengthening measures designed to train primary care staff in evidence-based interventions on a long-term basis. The project aims to provide universal mental health care coverage across six LMICs, including Ethiopia (see www.emerald-project.eu). Finally, a relatively large-scale project funded by the Grand Challenges Canada, the Biaber project, aims to look at scale-up of psychological interventions, primarily interpersonal therapy in a primary care setting. This study fills an important gap in the scale-up of services in low-income countries.

Specialist care provisions and research capacity are growing in parallel, with input from GMH partners. The Toronto Addis Ababa Psychiatry Project (TAAPP) (29), which began in 2003 in collaboration with the University of Toronto in Canada, has led to a substantial expansion of psychiatrists (from 10 to more than 50 now). Research capacity is also being developed through the on-going international research collaborations described here and a recently started PhD programme in collaboration with King's College London, UK. Amanuel Specialised Hospital (the only specialised mental health unit in the country), in collaboration with the University of Gondar and Jimma University, are conducting trainings for midlevel (master's-level) mental health practitioners, who will support some of the specialist and supervisory needs of the mental health service.

## Discussion

This article demonstrates three important issues: 1) the serious personal (and societal) consequences of the lack of accessible care (the treatment gap), 2) the life-transforming impact of provision of care, and 3) the potential to expand services to reach the population in need through affordable scale-up of care. The treatment gap, as demonstrated by the two case examples, is not an abstract concept but a concrete issue with a real impact on citizens. It leads to personal and family distress, significant financial cost, loss of human rights and dignity, and exploitation. Because of the structure of the care system, the impact on families is unmitigated and may even affect multiple generations, as exemplified by the first case. The situation faced by the pregnant woman in the first case example also demonstrates that the care of the centralised services is not geared to address complex needs. Often, in the effort to expand integrated care, tertiary services are neglected. It is essential that some services are able to provide complex care. The treatment gap, even with the additional service expansions, remains substantial. If this is extrapolated to the entire country of Ethiopia, millions of people with mental illness and their families endure the consequences of mental illness, as exemplified by these cases. Intellectual debates (30, 31) often have no resemblance to the real-life challenges that people with mental illness, their families, and even their communities face in low-income countries. Yet that the transformative care provided by the Butajira project is led by psychiatric nurses with limited training is a testimony to the fact that effective care can be provided at relatively low cost. The model of the psychiatric nursing programme that expanded care to 58 sites across Ethiopia is also a demonstration of the possibility of expanding effective care.

However, although achievable, it should be recognised that scaling up mental health services in low-income settings is a monumental task. We need to know more to understand the best approaches to take. In this regard, Ethiopia is also a good example. The complementary projects being conducted currently will provide evidence on the best approaches to take to provide safe, task-shared care for severe mental disorders (TASCS project); on how local integration should work (PRIME project); and on how system-level integration should look (EMERALD: www.emerald-project.eu). Ethiopia is also a very good example on what governments should do. The government has demonstrated its commitment to the scale-up of mental health care by providing policy guidance and by supporting scale-up through the mhGAP programme. Addis Ababa University, the leading research institution in the country, has also included mental health as one of the priority areas for research. However, integration alone (care by non-specialists and involvement of the informal sector) cannot be the solution. Specialist capacity for services and research is also essential, as shown in Ethiopia. Moreover, the work requires urgency and diligence on all sides: governments should push for achieving their targets within a set timeframe, health care professionals need to be guided by a sense of public service, and the community should be aware of and support local initiatives as well as demand the care it deserves. The urgency to expand system capacity, care staff capacity, and staff morale and well-being should be taken seriously. Along with integrated care, the pathway to patient care should also include expansion of specialist capacity to enable training, mentoring, supervision, and care. Attention should be paid to patients presenting to general medical settings with complex needs. These patients are likely to require specialist inputs akin to what would be a consultation liaison service in the West. It is safe and essential to fund mental health research and service delivery. Additionally, the focus of the current effort to scale up service is rightly on expanding coverage to the rural population. But cities are rapidly expanding. There is a large movement of population from the countryside to the cities. There seems to be a considerable change in the social fabric of the population, for example in terms of the volume of reliable social networks (32). This is also the trend in most parts of Africa. Therefore, developing evidence on the best approaches for the provision of care in the cities, for vulnerable mobile populations and urban slums, should not be neglected. In view of the huge expansion of medical schools during the past 20 years, revising and standardising the mental health curriculum to incorporate the mhGAP intervention guide would be useful. Mental health research and service delivery are at a crossroads. But no one should accept the status quo in the 21st century.

## References

[1] Koplan JP, Bond TC, Merson MH, Reddy KS, Rodriguez MH, Sewankambo NK (2009). Towards a common definition of global health. Lancet.

[2] Murray CJ, Vos T, Lozano R, Naghavi M, Flaxman AD, Michaud C (2012). Disability-adjusted life years (DALYs) for 291 diseases and injuries in 21 regions, 1990–2010: a systematic analysis for the Global Burden of Disease Study 2010. Lancet.

[3] Abdullahi H, Mariam DH, Kebede D (2001). Burden of disease analysis in rural Ethiopia. Ethiopian Med J.

[4] Patel V, Thornicroft G (2009). Packages of care for mental, neurological, and substance use disorders in low- and middle-income countries: PLoS Medicine Series. PLoS Med.

[5] Wang PS, Aguilar-Gaxiola S, Alonso J, Angermeyer MC, Borges G, Bromet EJ (2007). Use of mental health services for anxiety, mood, and substance disorders in 17 countries in the WHO world mental health surveys. Lancet.

[6] Thornicroft G (2007). Most people with mental illness are not treated. Lancet.

[7] Patel V, Maj M, Flisher AJ, De Silva MJ, Koschorke M, Prince M (2010). Reducing the treatment gap for mental disorders: a WPA survey. World Psychiatr.

[8] Alem A, Kebede D, Fekadu A, Shibre T, Fekadu D, Beyero T (2009). Clinical course and outcome of schizophrenia in a predominantly treatment-naive cohort in rural Ethiopia. Schizophr Bull.

[9] mhGAP-Ethiopia Working Group (2010). Mental Health Gap Action Programme in Ethiopia: final document.

[10] Shibre T, Negash A, Kullgren G, Kebede D, Alem A, Fekadu A (2001). Perception of stigma among family members of individuals with schizophrenia and major affective disorders in rural Ethiopia. Soc Psychiatry Psychiatr Epidemiol.

[11] Thornicroft G, Brohan E, Rose D, Sartorius N, Leese M (2009). Global pattern of experienced and anticipated discrimination against people with schizophrenia: a cross-sectional survey. Lancet.

[12] Assefa D, Shibre T, Asher L, Fekadu A (2012). Internalized stigma among patients with schizophrenia in Ethiopia: a cross-sectional facility-based study. BMC Psychiatry.

[13] Shibre T, Kebede D, Alem A, Negash A, Deyassa N, Fekadu A (2003). Schizophrenia: illness impact on family members in a traditional society – rural Ethiopia. Soc Psychiatry Psychiatr Epidemiol.

[14] Yin RK (1994). Discovering the future of the case study method in evaluation research. Eval Prac.

[15] Fidel R (1984). The case study method: a case study. LISR.

[16] Kebede D, Alem A, Shibire T, Deyassa N, Negash A, Beyero T (2006). Symptomatic and functional outcome of bipolar disorder in Butajira, Ethiopia. J Affect Dis.

[17] World Health Organization (2010). mhGAP intervention guide.

[18] Dua T, Barbui C, Clark N, Fleischmann A, Poznyak V, van Ommeren M (2011). Evidence-based guidelines for mental, neurological, and substance use disorders in low- and middle-income countries: summary of WHO recommendations. PLoS Med.

[19] Kebede D, Alem A, Shibre T, Negash A, Fekadu A, Fekadu D (2003). Onset and clinical course of schizophrenia in Butajira-Ethiopia – a community-based study. Soc Psychiatry Psychiatr Epidemiol.

[20] United Nations (2006). Convention on the rights of persons with disabilities.

[21] Teferra S, Shibre T, Fekadu A, Medhin G, Wakwoya A, Alem A (2012). Five-year clinical course and outcome of schizophrenia in Ethiopia. Schizophr Res.

[22] Fekadu A, Kebede D, Alem A, Fekadu D, Mogga S, Negash A (2006). Clinical outcome in bipolar disorder in a community-based follow-up study in Butajira, Ethiopia. Acta Psychiatr Scand.

[23] Teferra S, Shibre T, Fekadu A, Medhin G, Wakwoya A, Alem A (2011). Five-year mortality in a cohort of people with schizophrenia in Ethiopia. BMC Psychiatry.

[24] 
Shibre T, Medhin G, Teferra S, Wakwoya A, Berhanu E, Abdulahi A (2012). Predictors of carer-burden in schizophrenia: a five-year follow-up study in Butajira, Ethiopia. Ethiopian Med J.

[25] FMOH (2010). Health Sector Development Program IV 2010/11 – 2014/15.

[26] FMOH (2012). National Mental Health Strategy 2011–2015.

[27] Daar AS, Jacobs M, Wall S, Groenewald J, Eaton J, Patel V (2014). Declaration on mental health in Africa: moving to implementation. Glob Health Action.

[28] Lund C, Tomlinson M, De Silva M, Fekadu A, Shidhaye R, Jordans M (2012). PRIME: a programme to reduce the treatment gap for mental disorders in five low- and middle-income countries. PLoS Med.

[29] Alem A, Pain C, Araya M, Hodges B (2010). Co-creating a psychiatric resident program with Ethiopians, for Ethiopians, in Ethiopia: the Toronto Addis Ababa Psychiatry Project (TAAPP). Acad Psychiatry.

[30] Summerfield D (2008). How scientifically valid is the knowledge base of global mental health?. BMJ.

[31] Summerfield D (2013). “Global mental health” is an oxymoron and medical imperialism. BMJ.

[32] Fekadu A, Medhin G, Selamu M, Hailemariam M, Alem A, Giorgis TW (2014). Population level mental distress in rural Ethiopia. BMC Psychiatry.

